# Polyomic profiling reveals significant hepatic metabolic alterations in glucagon-receptor (GCGR) knockout mice: implications on anti-glucagon therapies for diabetes

**DOI:** 10.1186/1471-2164-12-281

**Published:** 2011-06-01

**Authors:** Jianxin Yang, Margit L MacDougall, Michael T McDowell, Li Xi, Ru Wei, William J Zavadoski, Mark P Molloy, John D Baker, Max Kuhn, Over Cabrera, Judith L Treadway

**Affiliations:** 1 Pfizer Global Research and Development, Groton, CT, USA; 2 Pfizer Global Research and Development, Cambridge, MA, USA; 3 Australian Proteome Analysis Facility, Macquarie University, Sydney, Australia; 4 Current Address: Neurosciences at Johnson & Johnson PRD, USA

## Abstract

**Background:**

Glucagon is an important hormone in the regulation of glucose homeostasis, particularly in the maintenance of euglycemia and prevention of hypoglycemia. In type 2 Diabetes Mellitus (T2DM), glucagon levels are elevated in both the fasted and postprandial states, which contributes to inappropriate hyperglycemia through excessive hepatic glucose production. Efforts to discover and evaluate glucagon receptor antagonists for the treatment of T2DM have been ongoing for approximately two decades, with the challenge being to identify an agent with appropriate pharmaceutical properties and efficacy relative to potential side effects. We sought to determine the hepatic & systemic consequence of full glucagon receptor antagonism through the study of the glucagon receptor knock-out mouse (Gcgr^-/-^) compared to wild-type littermates.

**Results:**

Liver transcriptomics was performed using Affymetric expression array profiling, and liver proteomics was performed by iTRAQ global protein analysis. To complement the transcriptomic and proteomic analyses, we also conducted metabolite profiling (~200 analytes) using mass spectrometry in plasma. Overall, there was excellent concordance (R = 0.88) for changes associated with receptor knock-out between the transcript and protein analysis. Pathway analysis tools were used to map the metabolic processes in liver altered by glucagon receptor ablation, the most notable being significant down-regulation of gluconeogenesis, amino acid catabolism, and fatty acid oxidation processes, with significant up-regulation of glycolysis, fatty acid synthesis, and cholesterol biosynthetic processes. These changes at the level of the liver were manifested through an altered plasma metabolite profile in the receptor knock-out mice, e.g. decreased glucose and glucose-derived metabolites, and increased amino acids, cholesterol, and bile acid levels.

**Conclusions:**

In sum, the results of this study suggest that the complete ablation of hepatic glucagon receptor function results in major metabolic alterations in the liver, which, while promoting improved glycemic control, may be associated with adverse lipid changes.

## Background

Glucagon is a 29 - amino acid hormone that is secreted by the α cells of the pancreas. Glucagon works in concert with insulin to maintain glucose homeostasis and acts to stimulate hepatic glucose production in response to hypoglycemia. The glucagon receptor is a 7-transmembrane spanning G-protein-coupled receptor that is coupled to G_s _and activates adenylate cyclase to increase intracellular levels of cAMP. In turn, this leads to activation of glycogenolytic and gluconeogenic pathways. Glucagon increases glycogenolysis and gluconeogenesis and decreases glycogenesis and glycolysis in a concerted fashion via multiple mechanisms [1].

Mice lacking the glucagon receptor gene (Gcgr^-/- ^mice) exhibit a phenotype of improved glucose tolerance with decreased glucose levels under both fed and fasted conditions compared to control mice, but they do not have overt hypoglycemia under these conditions. The mice appear normal, reach normal body weight, and have normal plasma insulin levels, but display elevated circulating glucagon levels and modestly elevated plasma cholesterol in both the fed and fasted state [2,3]. Evaluation of the liver profile revealed similar liver weights between the control and the Gcgr -/- animals. However, in the fed but not fasted state, hepatic glycogen levels increase by 65%, suggesting the Gcgr^-/-^mice do not mobilize glycogen as efficiently as wild-type or favor glycogenesis [3]. Other phenotypic changes in the Gcgr^-/- ^mice include reduced adiposity and pancreatic α-cell hyperplasia [2,3]. It is known that liver glucose metabolism serves a critical role in whole body glucose homeostasis with metabolism of glucose being primarily by glycolysis and the tricarboxylic acid (TCA) cycle. While the Gcgr^-/- ^mice have been well-characterized physiologically, we performed a comprehensive analysis of transcriptomic and proteomic changes in the liver of these animals, as well as metabolic profiling of the plasma, to more thoroughly understand the consequence of glucagon receptor ablation at the molecular level. Major biological alterations were seen in Gcgr^-/- ^animals affecting carbohydrate metabolism, lipid metabolism, and protein metabolism with many of the pathways being affected at both the mRNA and protein level.

## Results

### Transcriptomic and Proteomic analysis

There were eight animals in both the GCGR^-/- ^and wild-type cohorts. Five animals from each group were selected for transcript profiling based on their RNA quality. No outliers were found during principal component analysis (PCA) and correlation mapping analysis (data not shown). For proteomics analysis, seven animals from each group were randomized then analyzed using the isobaric tag for relative and absolute quantitation (iTRAQ) platform (see Additional file 1). A QC analysis by PCA and manual screening for blood proteins such as hemoglobin indicated significant blood contamination in wild-type animals M5 and M7 (data not shown); therefore, these animals were omitted from differential expression analysis. The threshold for significance was a false discovery rate (FDR) < 0.02 for both the transcript and protein data. From the transcript analysis, 899 genes were identified as differentially expressed in the livers of Gcgr^-/- ^versus their wild-type littermates. From the protein analysis, 86 proteins were identified as differentially expressed.

### Gene ontology (GO) analysis of transcriptomic and proteomic profiling data

To gain an understanding of the biological alterations in liver of Gcgr^-/- ^mice compared to wild-type, we examined transcriptomic and proteomic data using GO and traditional pathway analysis [4]. Both the transcript and protein results were imported into the GO tools AmiGo (v1.7), Ingenuity Pathway Analysis, and GeneGo MetaCore™. Enrichments in similar biological processes and pathways were observed in all cases. The top biological processes enriched using AmiGo are summarized in Table 1. Of the 899 transcripts, 438 mapped to "metabolic process", reflecting a 48.7% representation versus a 20.2% background frequency. Under metabolic process ontology, several key energy metabolic processes were enriched in the transcriptomic dataset, including carbohydrate metabolism (4% vs. 1.3%), lipid metabolism (8.8% vs. 2.2%), and protein metabolism (18% vs. 6.8%). Similar enrichment in metabolic processes was found in the proteomic dataset. Overall, the representation frequency in these metabolic pathways by proteins is higher than that by mRNA, which is likely due to bias of proteomic technology toward detecting highly expressed proteins such as metabolism-related enzymes in the liver.

**Table 1:** Top Metabolic Processes Enriched in Transcriptomic and Proteomic Profiling of the Gcgr^-/- ^Mouse Liver

		Adj. p-value	Sample frequency	Background frequency
**GO:0008152 metabolism Process**	mRNA	7.80E-80	438/899 (48.7%)	6938/34273 (20.2%)
	Protein	4.14E-29	70/86 (81.4%)	
**GO:0009058 biosynthetic process**	mRNA	2.32E-27	207/899 (23.0%)	3429/34273 (10.0%)
	Protein	3.67E-06	29/86 (33.7%)	
**GO:0009056 catabolic process**	mRNA	4.59E-10	63/899 (7.0%)	839/34273 (2.4%)
	Protein	1.97E-20	31/86 (36.0%)	
**GO:0005975 carbohydrate metabolic process**	mRNA	2.56E-05	36/899 (4.0%)	456/34273 (1.3%)
	Protein	1.71E-07	13/86 (15.1%)	
**GO:0016051 carbohydrate biosynthetic process**	mRNA	5.18E-04	11/438 (2.5%)	107/34273 (0.3%)
	Protein	4.38E-07	8/86 (9.3%)	
**GO:0016052 carbohydrate catabolic process**	mRNA	1.37E-01	8/438 (1.8%)	97/34273 (0.3%)
	Protein	2.14E-04	6/86 (7.0%)	
**GO:0006006 glucose metabolic process**	mRNA	1.14E-03	18/899 (2.0%)	161/34273 (0.5%)
	Protein	3.80E-07	9/86 (10.5%)	
**GO:0006629 lipid metabolic process**	mRNA	1.60E-22	79/899 (8.8%)	742/34273 (2.2%)
	Protein	4.54E-11	19/86 (22.1%)	
**GO:0008610 lipid biosynthetic process**	mRNA	1.12E-18	48/899 (5.3%)	324/34273 (0.9%)
	Protein	2.14E-01	6/86 (7.0%)	
**GO:0016042 lipid catabolic process**	Protein	2.41E-07	9/86 (10.5%)	153/34033 (0.4%)
**GO:0006631 fatty acid metabolic process**	mRNA	2.93E-06	18/438 (4.1%)	223/34273 (0.7%)
	Protein	6.77E-13	14/86 (16.3%)	
**GO:0008654 phospholipid biosynthetic process**	mRNA	1.29E-06	17/899 (1.9%)	93/34273 (0.3%)
**GO:0008203 cholesterol metabolic process**	mRNA	1.05E-07	17/899 (1.9%)	80/34273 (0.2%)
**GO:0006695 cholesterol biosynthetic process**	mRNA	1.17E-05	10/899 (1.1%)	30/34273 (0.1%)
**GO:0019538 protein metabolic process**	mRNA	4.58E-27	162/899 (18.0%)	2325/34273 (6.8%)
**GO:0006412 ribosome translation**	mRNA	8.82E-13	53/899 (5.9%)	347/34273 (1.0%)
**GO:0006519 cellular amino acid and derivative metabolic process**	mRNA	5.13E-20	36/438 (8.2%)	312/34273 (0.9%)
	Protein	1.76E-19	20/86 (23.3%)	
**GO:0006508 proteolysis**	mRNA	2.57E-04	39/899 (4.3%)	567/34273 (1.7%)

Although there is high consensus in GO analysis between the transcriptomic and the proteomic data, only 32 of the 86 altered proteins overlapped with the 899 altered genes (see Additional file 2 for details). This is a well-observed phenomenon caused by differences in detection sensitivity and profiling scope between transcriptomic and proteomic technologies, and disparity in the regulation and kinetics of mRNAs versus proteins. Nevertheless, for the 32 proteins and genes that overlap, there is a high correlation in their expression changes (R^2 ^= 0.8833, see Additional file 3 for details).

### Alterations in carbohydrate metabolism in Gcgr^-/- ^mouse liver

A detailed pathway analysis revealed that key genes involved in carbohydrate metabolism have altered expression at both the mRNA and protein levels in the liver of knockout animals. As shown in Table 2, genes related to glycogenesis and glycogenolysis, such as glucan (1, 4-alpha-), branching enzyme 1 (Gbe1) and glycogen phosphorylase (Pygl), were up-regulated, indicating an increased flux in glycogen metabolism. Many genes central to the regulation of glycolysis and gluconeogenesis had altered expression, as well. Glucokinase (Gck) mRNA, enolase (Eno1) protein, and pyruvate kinase (Pklr) protein were all up-regulated to a greater or lesser extent, indicating activation of liver glycolytic pathway in Gcgr^-/- ^mice. In contrast, two key enzymes catalyzing gluconeogenesis, fructose bisphosphatase 1 (Fbp1) mRNA/protein and phosphoenolpyruvate carboxykinase 1 (Pck1) mRNA were significantly down-regulated. In addition, many genes that convert amino acids into substrates for gluconeogenesis, such as Agxt, Gpt, Sds, and Got1 were down-regulated (see Table 3 for detail).

**Table 2:** Significantly altered mRNA and proteins related to carbohydrate metabolism

	*Transcriptomics*		*Proteomics*	
***Glycogenesis***	***Adj. p-value***	***Fold change***	***Adj. p-value***	***Fold change***

Glucokinase (Gck)	4.84E-03	1.8		
Glucan (1,4-alpha-), branching enzyme 1 (Gbe1)	3.03E-03	2.3	2.98E-05	1.5
***Glycogenolysis***				
Amylo-1,6-glucosidase, 4-alpha-glucanotransferase (Agl)	6.05E-03	1.8		
Protein phosphatase 1, regulatory (inhibitor) subunit 2 (Ppp1r2)	4.00E-03	1.4		
Glycogen phosphorylase, liver form (Pygl)			1.41E-03	1.2
***Glycolysis***				
Glucokinase (Gck)	4.84E-03	1.8		
Aldolase C, fructose-bisphosphate (Aldoc)	8.48E-03	1.9		
Alpha-enolase (Eno1)			1.62E-03	1.1
Pyruvate kinase, liver (Pklr)			3.28E-05	1.5
Fructose-bisphosphate aldolase B (Aldob)			2.59E-03	1.2
Glucose-6-phosphate isomerase (Gpi)			6.90E-05	1.2
Glyceraldehyde-3-phosphate dehydrogenase (Gapdh)			1.10E-03	-1.2
***Gluconeogenesis***				
Fructose bisphosphatase 1 (Fbp1)	3.35E-03	-1.9	4.26E-06	-1.4
Phosphoenolpyruvate carboxykinase 1, cytosolic (Pck1)	3.22E-03	-10.2		
***TCA Cycle-related***				
Pyruvate dehydrogenase complex component E2 (Dlat)	4.48E-03	1.8		
Pyruvate dehydrogenase kinase, isoenzyme 1 (Pdk1)	7.73E-03	2.0		
Pyruvate dehyrogenase phosphatase catalytic subunit 2 (Pdp2)	6.96E-03	-1.4		
Fumarate hydratase (Fh1)	2.02E-03	-1.6	3.78E-03	-1.2
Succinate dehydrogenase flavoprotein subunit, mitochondrial (Sdha)	3.05E-03	-1.3		
2-oxoglutarate dehydrogenase complex component E2 (Dlst)			2.53E-03	-1.2

**Table 3:** Significantly Altered mRNA and Proteins related to Amino Acid Catabolism

	*Transcriptomics*		*Proteomics*	
***Amino Acid Metabolism***	***Adj. p-value***	***Fold change***	***Adj. p-value***	***Fold change***

Alanine-glyoxylate aminotransferase (Agxt)*	1.02E-02	-1.8	1.82E-03	-1.2
Cystathionase (cystathionine gamma-lyase) (Cth)*	1.66E-02	-1.5	8.36E-04	-1.4
Glutamic-oxaloacetic transaminase 1, soluble (Got1)*	9.42E-03	-2.8	1.05E-05	-2.1
Glutamic-pyruvate transaminase (Gpt)*	1.42E-02	-1.6	9.90E-04	-1.3
Glutamic pyruvate transaminase 2 (Gpt2)*	1.52E-02	-1.5		
Ornithine aminotransferase (Oat)*	1.08E-03	-5.0	2.52E-06	-2.3
Prolyl 4-hydroxylase, beta polypeptide (P4hb)*	1.62E-02	-1.4		
Serine dehydratase (Sds)*	1.85E-02	-2.6		
Serine dehydratase-like (Sdsl)*	1.89E-02	-1.5		
Homogentisate 1,2-dioxygenase (Hgd)*^	4.28E-03	-1.7	2.41E-06	-1.4
**Aminoadipate-semialdehyde synthase (Aass)^**	1.00E-02	-1.5		
**3-hydroxyanthranilate 3,4-dioxygenase (Haao)^**	4.33E-04	-2.4	4.57E-05	-1.4
**Isovaleryl Coenzyme A dehydrogenase (Ivd)^**	3.81E-03	-1.8		
**Kynureninase (L-kynurenine hydrolase) (Kynu)^**	1.61E-02	-2.1		
**Tyrosine aminotransferase (Tat)^**	1.72E-02	-2.2		
**Tryptophan 2,3-dioxygenase (Tdo2)^**	3.03E-03	-1.9		
**Alanyl-tRNA synthetase (Aars)**	8.80E-03	-1.3		
**δ-aminolevulinate synthase 2 (Alas2)**	1.11E-02	-2.9		
**Aldehyde dehydrogenase 4 family, member A1 (Aldh4a1)**	1.28E-02	-1.4		
**Carbamoyl-phosphate synthetase 2, aspartate transcarbamylase, and dihydroorotase (Cad)**	1.99E-02	-1.3		
**Cytochrome P450, family 7, subfamily B, polypeptide 1 (Cyp7b1)**	3.45E-03	-3.1		
**Dopa decarboxylase (aromatic L-amino acid decarboxylase) (Ddc)**	1.02E-02	-1.9		
**Formiminotransferase cyclodeaminase (Ftcd)**	2.88E-03	-1.6	3.52E-03	-1.3
**Glycine N-methyltransferase (Gnmt)**	1.05E-02	-1.5		
**Sarcosine dehydrogenase, mitochondrial (Sardh)**			3.73E-03	-1.2
**Glucosamine-phosphate N-acetyltransferase 1 (Gnpnat1)**	1.11E-02	-1.2		
**Leucine aminopeptidase 3 (Lap3)**	3.45E-03	-1.4	6.01E-06	-1.2
**Methyltransferase like 7B (Mettl7b)**	2.02E-03	-1.6		
**4-hydroxyphenylpyruvate dioxygenase (Hpd)**			9.17E-04	-1.3
**Phenylalanine hydroxylase (Pah)**	1.70E-02	-1.2	3.07E-05	-1.5
**Branched-chain alpha-keto acid dehydrogenase complex component E2 (Dbt)**			2.29E-03	-1.3
**4-aminobutyrate aminotransferase, mitochondrial (Abat)**			1.10E-03	-1.2
***Urea Cycle & Metabolism of Amino Groups***				
**Mitochondrial ornithine transporter 1 (Slc25a15)**	9.92E-03	-1.4		
Argininosuccinate synthetase 1 (Ass1)*	1.62E-02	-1.5	1.25E-07	-1.6
**Argininosuccinate lyase (Asl)**			1.35E-03	-1.2
**Arginase 1 (Arg1)**			1.53E-03	-1.2
Glutamate-ammonia ligase, Glutamine synthetase (Glul)*			1.04E-03	1.3
**Carbonic anhydrase3 (C**a**3)**			3.78E-03	-1.18

*Involved in catalyzing amino acids for gluconeogenesis

^Involved in catalyzing amino acids for ketone bodies

Pyruvate, the product of glycolysis, is converted to acetyl-CoA through the action of pyruvate dehydrogenase. As shown in Table 2, mRNAs for two components of the pyruvate dehydrogenase complex (Dlat and Pdk1) were up-regulated. Meanwhile, the mRNA for pyruvate dehyrogenase phosphatase catalytic subunit 2 (Pdp2) was down-regulated. Interestingly, moderate down-regulation was observed in the mRNA or the proteins involved in the TCA cycle, including down-regulation of succinate dehydrogenase, fumarate hydratase, and a subunit of the α-keto-glutarate dehydrogenase complex. Taken together, there was a significant increase in glycolysis and a decrease in acetyl-CoA oxidation, leading to potential acetyl-CoA buildup and *de novo *lipogenesis in Gcgr^-/- ^mouse liver.

### Alterations in amino acid metabolism in the Gcgr^-/- ^mouse liver

Amino acid catabolism was one of the most affected metabolic processes in the Gcgr^-/- ^liver. As shown in Table 1, GO analysis shows that 8.2% of the significantly changed genes map to amino acid catabolic processes, whereas the background frequency in the whole genome is only 0.9%. Interestingly, the expression of these enzymes was universally down-regulated at mRNA and/or protein level in the knockout mice (Table 3). As mentioned above, enzymes involved in amino acid degradation to fuel gluconeogenesis, such as Agxt, Gpt, Got1, and Sds, were down-regulated. In addition, many other amino acid catabolic enzymes were down-regulated in the Gcgr^-/- ^liver, as well. For instance, expression of enzymes, such as Haao, Ivd and Aass, which break down ketogenic amino acids into acetoacetate or acetyl-CoA, are decreased (Table 3). Furthermore, enzymes involved in the capture and disposal of amino acid nitrogen were down-regulated. For example, the glucose-alanine cycle is primarily responsible for the transport of excessive nitrogen from muscles to the liver for urea synthesis while replenishing muscle glucose supply. A key enzyme in the glucose-alanine cycle, liver Glutamic-pyruvate transaminase (Gpt), converts alanine to pyruvate for oxidation or gluconeogenesis, while eliminating its amino-group for urea synthesis. In the Gcgr^-/- ^liver, Gpt's expression was down-regulated at both mRNA and protein level. In fact, several enzymes in the liver urea cycle, including Ass1, Asl, Arg1, and Slc25a15, were down-regulated. One role glutamine plays in the body is to collect and carry nitrogen from peripheral tissues to liver. Liver glutaminase 2 (Gls2) is expressed in periportal hepatocytes, where it catalyzes the release of ammonia from glutamine for urea synthesis. In the Gcgr^-/- ^liver, Gls2 mRNA was reduced by 3.4 fold (Table 3).

There are three metabolic fates for liver amino acids: oxidation for energy, conversion to glucose or ketone bodies, or as building blocks for new proteins. Although the first two pathways are inhibited in the Gcgr^-/- ^liver as described above, genes related to protein synthesis, especially those encoding ribosomal proteins, are up-regulated by an average of ~ 20% (Additional file 4). Thus, there is a modest increase of protein synthesis in the Gcgr^-/- ^liver.

### Alterations in lipid metabolism in the Gcgr^-/- ^mice

As described above, in the Gcgr^-/- ^liver, the concentration of acetyl-CoA might rise due to increased glycolysis and reduced activity of TCA oxidation. Accumulated mitochondrial acetyl-CoA can be transported into the cytosol and utilized for *de novo *fatty acid (FA) biosynthesis. Interestingly, FA biosynthesis pathway appeared to be up-regulated at mRNA and protein level in the knockout mice (Table 4). Transcripts for both the master transcription factor that regulates FA biosynthesis Srebf1 (aka Srebp1) and its chaperone protein, Scap, were increased significantly. In addition, the rate-limiting enzymes for FA biosynthesis, acetyl-CoA carboxylase α and ß (Acaca and Acacb), which catalyze the synthesis of malonyl-CoA from acetyl-CoA, were up-regulated by 2.6 and 1.8 fold, respectively (Table 4). The multifunctional enzyme FA synthase (Fasn), which catalyzes the synthesis of palmitate from acetyl-CoA and malonyl-CoA, also was up-regulated at both the mRNA and protein level. Furthermore, the malic enzyme (Me1), which catalyzes cytosolic malate to pyruvate and generates NADPH for FA synthesis, was up-regulated significantly. Other notable enzymes that were up-regulated include: Elovl 6, which extends palmitate (16:0) to stearate (18:0), Scd1, which catalyzes the first step in synthesis of oleate (18:1) from stearate, and Acot 3 and 13, which hydrolyze acyl-CoAs to their corresponding free FAs (Table 4). Interestingly, Elovl 2 and 3, which catalyze the elongation of very long FAs in the endoplasmic reticulum, are down-regulated in the Gcgr^-/- ^liver. Of note, although not changed at the transcript level, the citrate lyase protein, which converts mitochondria-originated citrate to acetyl-CoA for lipid synthesis in cytoplasm, showed moderate up-regulation (Table 4).

**Table 4:** Significantly Altered mRNA and Proteins related to Fatty Acid Metabolism

	*Transcriptomics*		*Proteomics*	
***Fatty acid synthesis***	***Adj. p-value***	***Fold change***	***Adj. p-value***	***Fold change***

Fatty acid synthase (Fasn)	7.80E-03	2.4	6.88E-06	1.7
Malic enzyme (Me1)	9.68E-03	2.6	3.36E-05	1.6
Acetyl-Coenzyme A carboxylase alpha (Acaca)	1.30E-03	2.6		
Acetyl-Coenzyme A carboxylase beta (Acacb)	1.89E-02	1.8		
Acyl-CoA synthetase medium-chain family member 5 (Acsm5)	1.94E-02	1.2		
Acyl-CoA synthetase short-chain family member 2 (Acss2)	4.10E-03	2.5		
Stearoyl-Coenzyme A desaturase 1 (Scd1)	7.80E-03	6.4		
Acyl-CoA thioesterase 13 (Acot13)	1.63E-02	1.3		
Acyl-CoA thioesterase 3 (Acot3)	1.38E-02	3.8		
Elongation of very long chain fatty acids-like 2 (Elovl2)	7.31E-03	-1.4		
Elongation of very long chain fatty acids -like 3 (Elovl3)	1.64E-02	-2.9		
ELOVL family member 6, elongation of long chain fatty acids (Elovl6)	5.84E-03	2.4		
Mitochondrial trans-2-enoyl-CoA reductase (Mecr)	1.15E-02	-1.2		
Sterol regulatory element binding transcription factor 1 (Srebf1)	1.51E-02	1.3		
SREBF chaperone (Scap)	3.97E-03	1.3		
Fatty acid binding protein 5, epidermal (Fabp5)	1.01E-02	3.7		
ATP-citrate lyase (Acly)			7.37E-04	1.2
Acyl-CoA binding protein (Dbi)			4.39E-06	1.4
Corticosteroid 11-beta-dehydrogenase isozyme 1 (Hsd11b1)			3.01E-03	1.4
***Fatty acid ß*-oxidation*in mitochondria***				
Carnitine palmitoyltransferase 1a, liver (Cpt1a)	7.22E-03	-1.6		
Carnitine palmitoyltransferase 2 (Cpt2)	2.88E-03	-1.3		
Acyl-Coenzyme A dehydrogenase, short/branched chain (Acadsb)	9.13E-03	-1.3		
Acetyl-CoA acyltransferase 2 (Acaa2)			1.13E-03	-1.3
3,2 trans-enoyl-Coenzyme A isomerase (Dci)			8.72E-04	-1.2
***Fatty acid oxidation in peroxisome***				
3-ketoacyl-CoA thiolase B, peroxisomal (Acaa1)			1.99E-03	1.6
Very long-chain acyl-CoA synthetase (Slc27a2)			2.22E-03	1.3
Acyl-CoA oxidase 1, palmitoyl (Acox1)			3.96E-04	1.3
Hydroxysteroid (17-beta) dehydrogenase 4 (Hsd17b4)			6.40E-04	1.2
Peroxisomal bifunctional enzyme (Ehhadh)			8.51E-04	1.8
2-hydroxyacyl-CoA lyase 1 (Hacl1)			2.42E-03	1.3
***Triglyceride content-related***				
Very low density lipoprotein receptor (Vldlr)	1.37E-02	3.7		
Lipoprotein lipase (Lpl)	4.51E-03	3.3		

FAs are catabolized into acetyl-CoA mainly through ß-oxidation in the mitochondria. The rate-limiting step for FA oxidation lies in the transport of activated FAs (acyl-CoAs) into the mitochondria by carnitine palmitoyltransferase 1 and 2 (Cpt1 and 2). In the Gcgr^-/- ^liver, these two enzymes were down-regulated moderately (Table 4). FAs with aliphatic tails longer than 22 carbons are first shortened in peroxisomes before being catabolized into acetyl-CoA in the mitochondria. Surprisingly, several peroxisome ß-oxidation enzymes, such as Acaa1, Slc27a2, and Acox1, were up-regulated moderately at the protein level in the Gcgr^-/- ^liver (Table 4). Interestingly, expression of the VLDL receptor and lipoprotein lipase (LPL), which are involved in FA uptake by peripheral tissues, were up-regulated in the Gcgr^-/- ^liver (Table 4). It would be worthwhile to check if these genes are also up-regulated in other tissues in future studies.

As acetyl-CoA is also the substrate for de novo cholesterol biosynthesis, we investigated whether this pathway was dysregulated in the Gcgr^-/- ^liver. As shown in Table 5, several key enzymes in the cholesterol synthesis pathway, including Acat2, Hmgcs, Hmgcr, Pmvk, Idi1, Fdps, and Lss, were all up-regulated between 1.3 to 3.0 fold at the transcript level. As mentioned above, Scap, which is involved in sterol-dependent post-translational regulation of Srebp activity, was up-regulated at the transcript level, although expression of Srebp2, the master transcription factor controlling cholesterol biosynthesis, was unchanged (data not shown). In addition, the cholesterol esterification enzyme, sterol O-acyltransferase2 (Soat), was up-regulated by ~ 2 fold. Furthermore, transcripts for the heterodimeric transporter Abcg-5/8, which is responsible for transferring cholesterol to canalicular bile, was up-regulated. Other notable genes involved in cholesterol transport, such as Abcg1, Npc2, Ca1, Apoa1, Cxcl16, and Scarb1, were up-regulated, as well (Table 5).

**Table 5:** Significantly Altered mRNA and Proteins related to Cholesterol and Bile Acid Metabolism

	*Transcriptomics*		*Proteomics*	
***Cholesterol synthesis***	***Adj. p-value***	***Fold change***	***Adj. p-value***	***Fold change***

Acetyl-Coenzyme A acetyltransferase 2 (Acat2)	8.35E-03	2.1		
Farnesyl diphosphate farnesyl transferase 1 (Fdft1)	7.22E-03	1.9		
Farnesyl diphosphate synthetase (Fdps)	1.72E-02	1.7		
3-hydroxy-3-methylglutaryl-Coenzyme A synthase 1 (Hmgcs1)	5.73E-03	1.6		
3-hydroxy-3-methylglutaryl-Coenzyme A synthase 2 (Hmgcs2)	5.73E-03	1.3		
3-hydroxy-3-methylglutaryl-Coenzyme A reductase (Hmgcr)	7.56E-03	3.0		
Isopentenyl-diphosphate delta isomerase (Idi1)	7.22E-03	2.4	4.46E-04	1.7
Lanosterol synthase (Lss)	6.52E-03	2.8		
NAD(P) dependent steroid dehydrogenase-like (Nsdhl)	1.38E-02	1.7		
Phosphomevalonate kinase (Pmvk)	1.23E-02	2.2		
Sterol O-acyltransferase 2 (Soat2)	4.33E-04	2.1		
SREBF chaperone (Scap)	3.97E-03	1.3		
***Cholesterol Transport***				
ATP-binding cassette, sub-family G (WHITE), member 5 (Abcg5)	6.99E-03	2.4		
ATP-binding cassette, sub-family G (WHITE), member 8 (Abcg8)	4.61E-03	1.8		
ATP-binding cassette, sub-family G (WHITE), member 1 (Abcg1)	2.88E-03	1.7		
Niemann Pick type C2 (Npc2)	1.13E-02	1.4		
Caveolin 1, caveolae protein (Cav1)	7.16E-03	4.6		
Apolipoprotein A-I (Apoa1)	3.45E-03	1.2		
Chemokine (C-X-C motif) ligand 16 (Cxcl16)	1.32E-02	1.4		
Scavenger receptor class B, member 1 (Scarb1)	6.98E-03	1.5		
*Bile Acid Synthesis*				
Cytochrome P450, family 7, subfamily B, polypeptide 1 (Cyp7b1)	3.45E-03	-3.1		
Cytochrome P450, family 8, subfamily b, polypeptide 1 (Cyp8b1)	9.22E-04	-2.2		
Cytochrome P450, family 27, subfamily A, polypeptide 1 (Cyp27a1)	7.86E-03	1.4		
Cytochrome P450, family 39, subfamily a, polypeptide 1 (Cyp39a1)	3.73E-03	2.1		
Alpha-methylacyl-CoA racemase (Amacr)	2.01E-03	-1.4		
Sterol carrier protein 2, liver (Scp2)	1.18E-02	-1.2		
Bile acid-CoA:amino acid N-acyltransferase (Baat)			6.89E-04	1.3
Cysteine sulfinic acid decarboxylase (Csad)			7.17E-05	1.9
Hydroxysteroid (17-beta) dehydrogenase 4 (Hsd17b4)			6.40E-04	1.7
*Bile Acid Transport*				
Sodium/bile acid cotransporter (Slc10a1)	7.40E-03	1.3		
ATP-binding cassette, sub-family B (MDR/TAP), member 11 (Abcb11)	3.04E-03	1.3		
ATP-binding cassette, sub-family C (CFTR/MRP), member 3 (Abcc3)	2.37E-03	1.6		
ATP-binding cassette, sub-family C (CFTR/MRP), member 4 (Abcc4)	8.85E-04	7.0		
Organic solute transporter beta (Ostྞ)	7.12E-03	1.5		

Bile acids (BAs) are synthesized from cholesterol through multiple enzymatic steps [5]. Expression of the key enzymes in this pathway did not show consistent changes at either transcript or protein level. As seen in Table 5, cyp27a1 and cyp39a1 showed moderate up-regulation, cyp7b1 and cyp8b1 were down-regulated, but the rate-limiting enzyme Cyp7A1 was unchanged (data not shown). Several BA transporters showed upregulation at the transcript level in the Gcgr^-/- ^liver. Slc10a1 (Ntcp), which reabsorbs BAs from the portal vein, was slightly up-regulated. Abcb11 (Bsep) and Abcc3, (Mrp3) which excrete BAs into the canalicular bile, were up-regulated moderately. Interestingly, Abcc4, which secretes BAs into systemic circulation as an alternative pathway, was up-regulated almost 7-fold. Ostβ and Abcc3, two other efflux transporters involved in the alternative secretion pathway, were up-regulated, as well (Table 5).

### Metabolomic profiling of the Gcgr^-/- ^mouse plasma

Plasma samples from the nine Gcgr^-/- ^mice and nine wild-type littermates were analyzed by targeted LC/MS/MRM profiling to identify changes in up to 210 small molecule metabolites. As seen in Table 6, the most dramatically changed category of metabolites belonged to that of amino acids and their derivatives. The concentrations of 16 amino acids in the plasma of knockout animals were increased by 1.3 to 9.6 fold. The levels of arginine and cysteine derivatives and urea cycle intermediates also were increased significantly in the plasma. Some nucleotides and nucleotide derivatives showed increased concentrations, as well.

**Table 6:** Significantly Changed Plasma Metabolites (Fold of KO/WT)

*Amino acids*	*Adj. P-value*	*Fold Change*		*Adj. P-value*	*Fold Change*
**Alanine**	0.00E+00	5.1	Phospho-tyrosine	1.23E-02	-1.5
**Arginine**	0.00E+00	3.9	Glutathione Oxidized	1.40E-03	-2.1
**Asparagine**	0.00E + 00	8.1	Kynurenic Acid	1.30E-03	-2.3
**Aspartate**	0.00E + 00	3.6	S-(5'-adenosyl)-L-homocysteine	6.40E-03	-3.4
**Cysteine**	0.00E + 00	2.7	***Nucleotides and derivatives***		
**Glutamate**	0.00E + 00	2.4	Guanine	0.00E + 00	2.3
**Glutamine**	0.00E + 00	2.9	Orotic Acid	0.00E + 00	1.6
**Glycine**	0.00E + 00	7.8	Pyridoxine	0.00E + 00	3.6
**Histidine**	0.00E + 00	4	UMP	6.60E-03	2.1
**Lysine**	0.00E + 00	2.9	Uridine	1.20E-03	1.7
**Methionine**	0.00E + 00	1.6	dCMP	6.10E-03	2.8
**Proline**	0.00E + 00	2.3	dUTP	1.03E-02	-1.3
**Serine**	0.00E + 00	8.7	***Bile Acids***		
**Threonine**	0.00E + 00	9.6	Cholic Acid	1.24E-02	244.3
**Tyrosine**	1.00E-04	1.7	Glycocholic Acid	1.69E-02	154.0
**Valine**	1.00E-04	1.3	***Vitamin and Cofactors***		
***Arg/urea cycle derivatives***			Choline	5.00E-04	-1.3
**a-keto-Glutarate**	0.00E + 00	2.3	5-Methyl-THF	1.00E-04	3.6
**Argininosuccinate**	7.00E-03	3.7	Ascorbic Acid	7.70E-03	-1.2
**Citrulline**	0.00E + 00	3.3	Biotin	1.95E-02	1.6
**L-NMMA**	0.00E + 00	4.3	Dihydrofolic Acid	0.00E + 00	5.3
**Ornithine**	0.00E + 00	5.4	Pantothenic Acid	0.00E + 00	1.7
**SDMA**	5.00E-04	1.7	Pyridoxal-5-phosphate	1.64E-02	1.5
***Met/Cys derivatives***			4-Pyridoxic_Acid	2.00E-04	2.0
**Cystathionie**	0.00E + 00	3.7	Betaine	0.00E + 00	2.8
**Homocysteine**	0.00E + 00	2.4	***Glycerol and derivatives***		
***Other AA derivatives***			Glycerate-3-P	5.00E-04	-2.2
**2-Aminodipic Acid**	0.00E + 00	5.4	Glyceraldehyde	6.40E-03	-1.3
**Dimethyl Glycine**	0.00E + 00	3.1	Glycerate-2-P	1.00E-04	-2.3
**Homoserine**	2.60E-03	3	Glycerol	0.00E + 00	-2.7
**Hydroxyproline**	1.40E-03	2.7	Phosphoenolpyruvate	2.00E-04	-2.3
**L-5-Hydroxytryptophan**	4.00E-04	2.2	***Carbohydrates***		
**Creatine**	0.00E + 00	2	Glucose	1.00E-04	-1.4
**N-Carbamyl-b-Alanine**	1.80E-03	1.7	Fructose	1.80E-03	-1.4
**Trimethylamine-N-Oxide**	1.90E-03	1.7	Lactose	0.00E + 00	-2.0
**GABA**	1.81E-02	1.5	UDP-Glucose	5.20E-03	-1.9

Glycerol and several metabolites in the glycolysis/gluconeogenesis pathways, including glycerate-2-p, glycerate-3-p, and phosphoenolpyruvate, showed decreases of ~ 2 fold in plasma concentration. As reported previously [2,3], plasma glucose level decreased by 1.4-fold. Plasma UDP-glucose, an activated form of glucose that is involved in the glycosyltranferase reactions in metabolism, decreased in parallel.

Finally, the levels of two bile acids, cholic acid and glycocholic acid, were increased dramatically (244 and 154 fold, respectively) in the Gcgr^-/- ^plasma (Table 6).

## Discussion and Conclusions

Glucagon signaling is a major counterregulatory hormone to insulin. It promotes glycogenolysis and stimulates gluconeogenesis in the liver, resulting in higher glucose output to the blood stream. In many T2DM patients, in addition to insulin signaling deficiency, glucagon secretion has been found to be abnormally elevated due to dysregulation, exasperating T2DM symptoms and progression [6,7]. Thus, attenuating glucagon signaling may provide a sound strategy to manage T2DM. Indeed, in preclinical studies, inhibition of the glucagon receptor with antagonistic small molecules, monoclonal antibodies, or antisense oligonucleotides leads to reduced plasma glucose, improved insulin sensitivity, and improved glucose tolerance [8-11]. Furthermore, genetic knockout of the mouse GCGR by homologous recombination resulted in significant reduction of blood glucose, improvement in glucose tolerance and insulin sensitivity, and resistance to diet-induced obesity [2,3]. Whereas these data provided strong rationale for targeting GCGR as a treatment of T2DM, some side effects have also been observed in mice with severe disruption of glucagon signaling, including increased plasma cholesterol concentration and enlarged pancreas due to α-cell hyperplasia (2, 3, 8, 10]. The current studies were designed to profile mouse liver mRNA, protein levels, and plasma metabolite levels to identify changes that underlie the beneficial, as well as unwanted effects, caused by GCGR knockout.

Our data showed that GCGR knockout leads to a marked down-regulation of genes and proteins associated with liver gluconeogenesis and a modest up-regulation of those involved in glycolysis (Table 2). This is not unexpected as glucagon, in countering insulin, normally promotes expression of gluconeogenesis pathway genes and inhibits those of glycolytic pathway. The severe inhibition of liver gluconeogenesis and the modest increase of liver glycolysis likely contributed most to the improved glucose control in Gcgr^-/- ^mice that has been published previously [2,3]. The mRNA of enzymes involved in glycogenesis were modestly increased as expected from loss of GCGR signaling, but paradoxically, the genes and proteins involved in glycogenolysis were also up-regulated (Table 2).

An important observation in the current study is the wide-spread reduction of transcripts and proteins of enzymes involved in amino acid catabolism in the Gcgr^-/- ^liver (Table 3). These include enzymes responsible for converting amino acid carbon skeletons into substrates for gluconeogenesis and ketone body production, e.g. Agxt, Gpt, Aass, and Haoo. Protein synthesis-related genes, on the other hand, were up-regulated only modestly at the transcriptional level in the Gcgr^-/- ^liver (Additional file 4). As a result, blood concentration of amino acids and their derivatives increased significantly (Table 6). Whereas the scope and degree of the transcriptional dysregulation of amino acid metabolism in the current study were surprising, this could be attributed to the net effect of up-regulating insulin action while down-regulating glucagon action in the knockout mice. It is a well known fact that glucagon promotes, while insulin inhibits, amino acid catabolism to provide substrates for gluconeogenesis, ketogenesis, or direct energy source by oxidation. Such gross regulation of these enzymes at the transcriptional level, by either insulin or glucagon, has not been published previously. In fact, transcriptional regulation of amino acid catabolism has not been completely illustrated as these pathways are chiefly regulated by substrate availability and allosteric mechanisms [12]. The dramatic transcriptional alterations of these pathways may reflect an adaptive response to innate loss of glucagon signaling in the Gcgr^-/- ^liver. Further studies are needed to understand the complex regulatory mechanism leading to these changes.

Another important observation in this study relates to altered lipid metabolism in the Gcgr^-/- ^liver. There is a marked increase of transcripts and proteins for the key enzymes involved in the biosynthetic pathways of fatty acids and cholesterol (Table 4 and 5). As a classical function of insulin, in opposition to glucagon, is to promote fatty acid synthesis from excessive glucose [12], it is consistent that fatty acid biosynthesis is up-regulated in the Gcgr^-/- ^liver where insulin signaling dominates. Multiple lines of evidence have indicated that the transcription factor sterol regulatory element-binding protein (Srebp)-1c mediates insulin-induced stimulatory effect on fatty acid biosynthesis genes [13-15]. Indeed, our data showed that the transcripts for Srebp-1 and its chaperone Srebp cleavage-activating protein (Scap) were both up-regulated 1.3 fold in the Gcgr^-/- ^liver (Table 4).

As alluded to above, transcripts of key genes involved in the *de novo *cholesterol biosynthesis such as HMG CoA reductase were increased for ~ 2 fold (Table 5) in the Gcgr^-/- ^liver. The expression of these genes has been shown to be controlled by the transcription factor Srebp2 in a sterol-dependent manner [16]. In contrast to Srebp1, expression of Srebp2 has not been shown to be regulated by insulin or glucagon [17,18]. This observation is confirmed in the current study as SREBP2 transcript was unchanged in the knockout mice (data not shown). Srepb2 is synthesized first as inactive precursor bound to the ER membrane via two transmembrane domains. Upon cholesterol depletion, it is escorted by the cholesterol-sensing and escorting protein Scap to the Golgi apparatus, where the N-terminal domain is released from the membrane via proteolysis. The N-terminal domain, designated as nSrebp2, then translocates to nucleus and activates the transcription of its target genes [16]. As noted above, SCAP expression is up-regulated in the Gcgr^-/- ^liver (Table 4). Thus, even though the level of full-length Srebp2 is not changed in these hepatocytes, more nSrepb2 could be generated and translocated to the nucleus, where it could stimulate the transcription of key cholesterol synthesis genes. Further studies are needed to fully understand the mechanism underlying the gross transcriptional upregulation of cholesterol synthesis pathway in the Gcgr^-/- ^liver.

In addition to the transcriptional up-regulation, fatty acid and cholesterol biosynthesis in the Gcgr^-/- ^liver may also be boosted by the potential accumulation of their common substrate acetyl-CoA, due to increased glycolysis, reduced oxidation via the TCA cycle, and inhibited gluconeogenesis (Table 2). Furthermore, our data showed that enzymes regulating mitochondrial β-oxidation of fatty acids were down-regulated at the transcriptional level, which is in line with a recently published biochemical study of fatty acid oxidation using 1-^14^C-palmitate labeling in the knockout mice [19]. Taken together, one would expect the plasma levels of free fatty acids (FFA), TG, and cholesterol to rise in Gcgr^-/- ^mice. Indeed, several publications, as well as our data (not shown), have shown that plasma cholesterol and/or LDL-C increase significantly in the knockout mice [2,3]. With regard to blood levels of FFA and TG, earlier reports indicated that there was little change in Gcgr^-/- ^mice compared to the wild-type littermates under fed or fasted condition [2, 3, and 20]. However, a recent study by Longuet and colleagues demonstrated that under prolonged fasting (16 hr), plasma FFA, TG, and liver VLDL secretion all rose significantly in Gcgr^-/- ^mice [19]. This discrepancy merits further study.

A surprising finding in the current study is the highly elevated level of cholic acid and glycocholic acid in the blood of Gcgr^-/- ^mice (Table 6). Bile acids (BAs) are synthesized from cholesterol in hepatocytes through a multistep enzymatic process and are secreted into the bile via the bile salt export pump ABCB11. BAs are released from the gall bladder into the intestinal lumen upon feeding to facilitate digestion of lipids. The majority of secreted BAs are reabsorbed efficiently into portal blood by specific transporters in the terminal ileum, and then taken up by hepatocytes through the action of basolateral uptake transporters, thus fulfilling an "enterohepatic cycle" [5].

Although the transcription of critical genes involved in liver BA synthesis, such as the rate-limiting enzyme Cyp7a1, were not up-regulated in the Gcgr^-/- ^liver (data not shown), BA production most likely increased in these mice owing to elevated level of cholesterol, the substrate for BA synthesis. BA excretion to the canalicular bile and uptake from the portal vein is predicted to increase moderately based on up-regulation of the transporter genes associated with these processes (Table 5). Hepatic BAs can be secreted to the systemic circulation via alternative basolateral efflux transporters and disposed of in urine [21]. In adaptive response, this alternative secretive pathway is up-regulated dramatically in mice when hepatic BAs accumulate as a result of bile duct ligation or suppression of ABCB11 expression in FXR knockout mice [22]. Interestingly, three of these alternative efflux transporters: Abcc3, Abcc4, and Ostβ are up-regulated by 1.6, 7.0, and 1.5 fold at the transcript level, respectively, in Gcgr^-/- ^mice (Table 5). As a result, BA excretion to the systemic circulation via these alternative transporters could increase dramatically in these mice, leading to the higher plasma level of BAs observed (Table 6). Thus, in Gcgr^-/- ^mice, hepatic BA production probably rises due to increased cholesterol synthesis, and BA excretion to bile and urine via blood is predicted to increase in adaptive responses.

It has been reported that plasma levels of glucagon-like peptide-1 (GLP1), as well as glucagon, were significantly elevated in Gcgr^-/- ^mice [2,3]. The increase in circulating GLP1 was caused, at least partly, by increased production and processing of the preproglucagon mRNA from hyperplasic alpha cells in these animas [3]. As BAs were recently found to be able to stimulate GLP1 secretion from enteroendocrine cells via the TGR5 receptor [5,21], it is tempting to speculate that elevated BAs may partially contribute to the increase of plasma GLP1 in these animals. Further studies are warranted to investigate this possibility as well as potential effects of elevated BAs on glucose homeostasis, lipid metabolism, and thermogenesis.

In summary, genetic knockout of the glucagon receptor in mice brought about significant metabolic changes in the liver. These include up-regulation of glycolysis, severe inhibition of gluconeogenesis and amino acid degradation, marked reduction of plasma glucose, and increased levels of plasma amino acids. Meanwhile, there is evidence indicating the up-regulation of fatty acid and cholesterol biosynthesis pathways and bile acid generation in these mice. In assessing the significance of these findings to anti-GCGR therapies for T2DM, we are mindful of the limitations to the current study. First, the tissues were collected for analysis when the animals were in the fed state. As many transcripts and proteins were likely turned over rapidly in response to fasting and feeding, our observation might not reflect the full metabolic status that exists in the fasted state. While glucagon signaling may contribute to the metabolic disorder in T2DM throughout the day, its most profound effects are thought to occur in the postprandial state. Second, our findings reflect the profound metabolic changes in animals with genetic ablation of the GCGR, while pharmaceutical inhibition of GCGR is unlikely to result in a complete and constant blockade of GCGR signaling. As inhibition of the glucagon signaling pathway presents an attractive therapeutic strategy for T2DM, the challenge will be to design drugs with optimal pharmacokinetic and pharmacodynamic properties that render glucose-lowering benefits while avoiding the potential side effects of lipogenesis.

## Methods

### Animals

Glucagon receptor knockout mice were previously generated by homologous recombination in embryonic stem cells on a DBA/1LacJ background [2]. Mice were bred and genotyped at Pfizer and housed in our animal facility. Animals were fed ad libitum with free access to water and maintained on a 12-h light, 12-h dark cycle under controlled temperature (20-22°C) and humidity (40-60%). All protocols were approved by the Institutional Animal Care and Use Committees.

### Sample collection

At 3-4 months of age, fed male wild-type and homozygous knockout animals were euthanized via carbon dioxide inhalation. Blood was collected by cardiac puncture into EDTA containers, and plasma was prepared and stored at -80°C until use. Livers were perfused with ice-cold PBS via the portal vein and two liver samples (200 mg each, one for RNA isolation, and the other for proteomics) were taken, flash frozen in liquid nitrogen, pulverized, and stored at -80°C.

### RNA isolation

Approximately 50 mg of the pulverized liver tissue was used to prepare total RNA by extracting in Trizol (Invitrogen, Carlsbad, CA, USA) followed by purification over a Qiagen RNeasy mini column as recommended by the manufacturer (Qiagen Inc., Valencia, CA, USA). RNA was purified further using Agencourt RNA Clean magnetic beads (Beckman Coulter, Inc.). The quantity and purity of the RNA was determined by absorbance at 260 nm and 260 nm/280 nm absorbance ratio, respectively. Each of the total RNA preparations was assessed individually for RNA quality based on the 28S/18S ratio and the RNA Integrity Number (RIN) measured on an Agilent 2100 Bioanalyzer system using the RNA 6000 Nano LabChip Kit.

### Microarray Expression Profiling

Affymetric analysis was performed at GeneLogic (Gaithersburg, MD) as follows. cDNA was synthesized using 1-2 ug of high quality total RNA. Supercript II™ (Invitrogen) was used to reverse-transcribe the mRNA in the presence of a T7 containing oligo dT24 primer followed by second strand synthesis as recommended by Affymetrix. cDNA was purified using Agencourt RNAClean magnetic beads and used as a template for in vitro transcription using the GeneChip^® ^One-Cycle Labeling Kit from Affymetrix. cRNA was purified using Agencourt magnetic beads. The quantity and purity of the cRNA was determined by absorbance at 260 nm and 260/280 absorbance ratio respectively. The quality of the cRNA was evaluated by assessing the size distribution of the cRNA using a 1.25% MOPS gel.

The labeled cRNA was fragmented as recommended by Affymetrix, and 10 μg was added to a hybridization cocktail prior to loading onto individual MOE430 2.0 GeneChip^®^. The microarrays were hybridized at 45°C for 16- 24 hours, washed and stained on an Affymetrix FS450 fluidics station according to manufacturer recommendations. The microarrays were scanned on a GeneChip^® ^Scanner 3000. GeneChip^® ^analysis was performed using Microarray Analysis Suite version 5.0 to generate expression values. All of the genes represented on the GeneChip^® ^were normalized globally and scaled to an average signal intensity of 100.

Array data quality was evaluated using a proprietary high-throughput application that assesses the data against multiple objective standards including 5'/3' GAPDH ratio, signal/noise ratio, and background, as well as other metrics (e.g. Negative PM-MM,% Present) and a visual inspection of the chip image for surface defects. Only samples that met preset standards were included in this analysis. Principal component analysis and correlation mapping was used to identify outlier samples that were removed from sample sets. Expression values were determined using the robust multichip average (RMA) method. Analysis of differential expression was conducted on a per-probeset basis.

### Proteomic Analysis

#### Sample preparation

Perfused and pulverized liver tissue from each animal was solubilized in lysis buffer (20 mM Na-Hepes, 0.1%(v/v) SDS, 150 mM NaCl, 1 mM EDTA, 1 mM NaVO_3_, 10 mM NaF, 1 mM EGTA, 1 mM PMSF), and protein concentrations were determined using the Bradford Protein Assay. The reference sample was made by combining 50 μg of total protein from each of the 16 samples available for analysis.

#### iTRAQ

Stock reagents and buffers were obtained in kit form from Applied Biosystems. All steps were done in the same tubes for each sample. Each sample (100 μg, total protein) was reduced and alkylated with 200 mM MMTS (Methyl methane Thiosulfonate). The samples were digested with trypsin for 16 hours at 37°C. Each digested sample was dried, resuspended in 0.5 M TEAB (Triethylammonium bicarbonate), and labeled with an iTRAQ reagent as described in Song et al [23] and as indicated in the Additional file 1. Each sample was analyzed twice using different iTRAQ reagent channels.

#### Chromatography and Data Acquisition

The Agilent 1100 nanoLC system (Agilent) and QStar Elite MS/MS system (Applied Biosystems) were used for nanoLC electrospray MS/MS. The sample solution was resuspended in 100 μL of loading/desalting solution (0.1% (v/v) trifluoroacetic acid, 2% (v/v) acetonitrile in water). A 40 uL aliquot of the resuspended solution was loaded onto a reverse phase peptide Captrap (Michrom Bioresources) and desalted with the desalting solution at 10 μL per minute for 15 minutes. After desalting, the trap was switched on line with a 150 μm × 10 cm C18 3 μm 300A ProteCol column (SGE). The buffer solutions were as follows: in Channel 1 was 0.1% (v/v) formic acid and 2% (v/v) acetonitrile in water; Channel 2A was and 0.1% (v/v) formic acid in water; Channel 2B was 90% acetonitrile, 9.9% (v/v) water, and 0.1% (v/v) formic acid. The buffer in Channel 2B was increased from 5% to 100% at 500nL per minute over a 93 minutes period in three linear gradient steps to elute peptides from the column. After peptide elution, the column was cleaned with Channel 2B solution for 20 minutes and then re-equilibrated with 95% Channel 2A for 8 minutes before next sample injection. The reverse phase nanoLC eluent was subject to positive ion nanoflow electrospray analysis in an information dependent acquisition mode (IDA). In IDA mode, a TOFMS survey scan was acquired (m/z 380-1600, 0.5 second), with the three most intense multiply charged ions (counts >70) in the survey scan sequentially subjected to MS/MS analysis. MS/MS spectra were accumulated for 2 seconds in the mass range m/z 100-1600 with a modified Enhanced All Q2 transition setting favoring low mass ions so that the reporting iTRAQ tag ion (113, 114, 115, 116, 117, 118, 119 and 121) intensities were enhanced for quantification.

#### Data Processing

The experimental nanoLC ESI MS/MS data were submitted to ProteinPilot (Applied Biosystems, version 3.0) for data processing. Paragon method was used in Thorough ID search effort with Biological modifications selected in ID Focus. The correction factors for the iTRAQ reagents (113, 114, 115, 116, 117, 118, 119 and 121) were entered in the iTRAQ Isotope Correction Factors table. The detected protein threshold (unused ProtScore) was set as larger than 1.3 (better than 95% confidence). For the mouse liver samples, the database used was SwissProt (version 54.6) with Mus musculus species selected.

#### Data Analysis

The output from the iTRAQ experiment was reduced to a table of reporter ion intensities relative to the intensity of the internal standard for each peptide across the multiplexed samples constituting a run. The intensities were adjusted for the specific isotope abundances of the iTRAQ reagents used in the experiment. The peptide sequence (and modifications) and protein ID associated with the peptide are also reported. Additional metadata including measured and theoretical molecular weight, ion and ID quality metrics were recorded.

An intuitive quantification tool was developed for the analysis the iTRAQ data. This procedure is quite robust and facilitates outlier determination by recognizing that each peptide ratio actually represents a simple x-y data pair in a given run. When the whole collection of x-y pairs for a given protein is plotted, linear regression yields a line the slope of which is the relative concentration of the protein of interest. For each protein, the slope, R^2 ^value and number of unique peptides was returned. This method allowed regression outliers to be easily identified, visually highlighted, and removed from the analysis. Since regression also weights higher intensity pairs more than lower intensity ones, greater influence is properly given to these points.

With the collection of two technical replicates in independent runs for each sample, the opportunity to compare these data for consistency was facilitated. Under ideal circumstances, the direct comparison of proteins from technical replicates should form a straight line of slope one. If the line is straight but the slope is different from one, this is indicative of bias in the reagents and subsequent reporter ions. The bias in the reporter ion channels was adjusted so that within a run, using all the high confidence protein ratios (based on R^2 ^> 0.9 and minimum number of unique peptides > = 3), that the mean fold change is 1.0. When applying this procedure, comparisons of technical replicates between independent runs indicate a slope of 1.

Following the bias adjustment and the QC of ratio outliers, run statistics were captured in an Excel spreadsheet. Protein ratios and associated metadata (R^2^, number of unique peptides, total number of ions, and maximum intensity of reported ions) were captured. In these studies, the filtering was set to a minimum R^2 ^value >0.70 with a minimum of two unique peptides with four observations. After filtering, technical replicates are averaged to generate a composite ratio. For cases where one run captures a protein ratio, and a second run does not, the single value is used for the composite. Additionally, significantly mismatched protein ratios between replicates were captured and filtered out of subsequent analysis.

### Plasma Metabolite Profiling

Detailed experiment conditions were described in a published methodology paper by Wei et al. [24]. Briefly, 37.5 μL of plasma was mixed with 75 μL of extraction buffer (80/20 ethanol/water, v/v, and 0.1% (v/v) formic acid), incubated at 4°C for 2 h, centrifuged at 14,000 g at 4°C for 15 min. 50 μL of supernatant was transferred into a 96-well PCR plate, and dried down under N_2_. The dry metabolite extract was reconstituted into 50 μL of water containing internal standards before analysis. Two hundred five endogenous metabolites, representative of important and relevant metabolic pathways, were analyzed by a 10 minute LC/MS/MRM method to determine significant changes in the plasma metabolome due to GCGR knockout.

### Statistical analysis

Analysis of differential expression between GCGR knockout and wild-type mice was conducted for each transcript, protein and metabolite by Analysis of Variance (ANOVA) t-test. The ANOVA t-statistics were adjusted using the moderated t-statistic method of Smyth (2004). False discovery rate corrections were applied to the resulting p-values using the Benjamini and Hochberg procedure [25]. Fold-changes were calculated using the group means produced by the ANOVA models. Principal component analysis and correlation mapping was used to identify outlier samples that were removed from sample sets.

## Authors' contributions

JY - Pathway analysis & data interpretation, lead writer of manuscript, MLM - Experiment planning and execution, manuscript writing, MTM - Proteomics analysis and interpretation, manuscript writing, LX -Statistical and data analysis, RW - Metabolomics data generation, WJZ - In vivo experiment, MPM - Proteomic analysis and interpretation, JDB - Proteomics data processing, MK - RNA profiling statistical support and consultation, OC - Biological context and manuscript review, JLT - Experimental design, manuscript writing and review. All authors read and approved the final manuscript.

## Supplementary Material

Additional file 1 **iTRAQ labeling scheme. Wild-type and knockout animals along with the internal standard (I.S.) were randomized for iTRAQ analysis**. For proteomics analysis, seven animals from each group were randomized then analyzed using the isobaric tag for relative and absolute quantitation (iTRAQ) platform Click here for file

Additional file 2 **Genes with overlapped expression changes between mRNA and proteins**. The table summarized genes with significant expression changes at both mRNA and protein levels Click here for file

Additional file 3 **Correlation analysis between mRNA and Protein Changes**. Linear regression analysis demonstrated correlation between mRNA and protein expression changes Click here for file

Additional file 4 **Significantly Changed mRNA related to Protein Translation**. The table summarized significantly changed mRNA related to protein translation in the GCGR KO liver. Click here for file
