# Supplementary material for: Polyomic profiling reveals significant hepatic metabolic alterations in glucagon-receptor (GCGR) knockout mice: implications on anti-glucagon therapies for diabetes
Source: BMC Genomics. 2011 Jun 1;12:281. doi: 10.1186/1471-2164-12-281 (PMC3130710; doi:10.1186/1471-2164-12-281)
Supplement: Additional file 1 — iTRAQ labeling scheme. Wild-type and knockout animals along with the internal standard (I.S.) were randomized for iTRAQ analysis. For proteomics analysis, seven animals from each group were randomized then analyzed using the isobaric tag for relative and absolute quantitation (iTRAQ) platform [file 1471-2164-12-281-S1.DOC]

**Additional file 1:** iTRAQ Labeling Scheme.

Wild-type and knockout animals along with the internal standard (I.S.) were randomized for iTRAQ analysis.

|  | **iTRAQ Reagent** | | | | | | | |
| --- | --- | --- | --- | --- | --- | --- | --- | --- |
| **Experiment** | 113 | 114 | 115 | 116 | 117 | 118 | 119 | 121 |
| Run 1 | WT-M4 | KO-M2 | I.S. | KO-M3 | WT-M5 | KO-M4 | WT-M6 | KO-M5 |
| Run 2 | WT-M7 | KO-M6 | WT-M8 | I.S. | KO-M7 | WT-M9 | KO-M8 | WT-M10 |
| Run 3 | WT-M8 | WT-M10 | KO-M8 | Wt-M9 | KO-M7 | WT-M7 | I.S. | KO-M6 |
| Run 4 | KO-M5 | I.S. | WT-M6 | KO-M4 | WT-M4 | KO-M3 | WT-M5 | KO-M2 |
