# Supplementary material for: Polyomic profiling reveals significant hepatic metabolic alterations in glucagon-receptor (GCGR) knockout mice: implications on anti-glucagon therapies for diabetes
Source: BMC Genomics. 2011 Jun 1;12:281. doi: 10.1186/1471-2164-12-281 (PMC3130710; doi:10.1186/1471-2164-12-281)
Supplement: Additional file 2 — Genes with overlapped expression changes between mRNA and proteins. The table summarized genes with significant expression changes at both mRNA and protein levels [file 1471-2164-12-281-S2.DOC]

**Additional file** 2: Genes with Overlapped Expression Changes between mRNA and Proteins

|  | **Transcriptomics** | | **Proteomics** | |
| --- | --- | --- | --- | --- |
| **Description** | Adj p-value | Fold Change | Adj. p-value | Fold Change |
| Alanine-glyoxylate aminotransferase (Agxt) | 1.02E-02 | -1.82 | 1.82E-03 | -1.16 |
| Argininosuccinate synthetase 1 (Ass1) | 1.62E-02 | -1.45 | 1.25E-07 | -1.56 |
| Biliverdin reductase B (flavin reductase (NADPH)) (Blvrb) | 4.62E-03 | 1.63 | 6.84E-05 | 1.43 |
| Carboxylesterase 1 (Ces1) | 3.45E-03 | 1.95 | 5.86E-07 | 1.57 |
| Carboxylesterase 3 (Ces3) | 7.22E-03 | 1.87 | 1.11E-06 | 1.54 |
| Cystathionase (cystathionine gamma-lyase) (Cth) | 1.66E-02 | -1.46 | 8.36E-04 | -1.37 |
| Peroxisomal bifunctional enzyme (Ehhadh) | 1.28E-02 | 2.00 | 8.51E-04 | 1.75 |
| Epoxide hydrolase 2, cytoplasmic (Ephx2) | 1.99E-02 | -1.33 | 2.91E-04 | -1.22 |
| Fatty acid synthase (Fasn) | 7.80E-03 | 2.38 | 6.88E-06 | 1.70 |
| Fructose bisphosphatase 1 (Fbp1) | 3.35E-03 | -1.93 | 4.26E-06 | -1.43 |
| Glucan (1,4-alpha-)branching enzyme 1 (Gbe1) | 3.03E-03 | 2.29 | 2.98E-05 | 1.51 |
| Glyoxalase domain containing 4 (Glod4) | 1.25E-02 | 1.19 | 2.07E-04 | 1.13 |
| Glycine N-methyltransferase (Gnmt) | 1.05E-02 | -1.45 | 5.12E-04 | -1.32 |
| Glutamate oxaloacetate transaminase 1, soluble (Got1) | 9.42E-03 | -2.84 | 1.05E-05 | -2.08 |
| Glutamic pyruvic transaminase, soluble (Gpt) | 1.42E-02 | -1.57 | 9.90E-04 | -1.28 |
| Glutathione S-transferase, mu 1 (Gstm1) | 6.59E-03 | 2.06 | 5.69E-06 | 2.01 |
| Glutathione S-transferase, pi 1 (Gstp1) | 3.17E-03 | -1.32 | 5.43E-06 | -1.54 |
| 3-hydroxyanthranilate 3,4-dioxygenase (Haao) | 4.33E-04 | -2.41 | 4.57E-05 | -1.39 |
| Hydroxyacyl glutathione hydrolase (Hagh) | 1.08E-02 | -1.25 | 6.94E-06 | -1.22 |
| Homogentisate 1, 2-dioxygenase (Hgd) | 4.28E-03 | -1.69 | 2.41E-06 | -1.39 |
| Isopentenyl-diphosphate delta isomerase (Idi1) | 7.22E-03 | 2.38 | 4.46E-04 | 1.68 |
| Leucine aminopeptidase 3 (Lap3) | 3.45E-03 | -1.37 | 6.01E-06 | -1.20 |
| NADP-dependent malic enzyme (Me1) | 9.68E-03 | 2.57 | 3.36E-05 | 1.55 |
| Nudix -type motif 7 (Nudt7) | 8.16E-03 | -1.74 | 3.06E-05 | -1.49 |
| Ornithine aminotransferase (Oat) | 1.08E-03 | -4.98 | 2.52E-06 | -2.27 |
| Phenylalanine hydroxylase (Pah) | 1.70E-02 | -1.21 | 3.07E-05 | -1.47 |
| Peptidylprolyl isomerase A (Ppia) | 7.56E-03 | 1.09 | 6.12E-04 | 1.15 |
| Selenium binding protein 1 (Selenbp1) | 7.73E-03 | -1.94 | 5.76E-04 | -1.43 |
| Transketolase (Tkt) | 7.28E-03 | 1.42 | 8.27E-08 | 1.42 |
| Formimidoyltransferase-cyclodeaminase (Ftcd) | 2.88E-03 | -1.55 | 3.52E-03 | -1.30 |
| Fumarate hydratase, mitochondrial (Fh1) | 2.02E-03 | -1.63 | 3.78E-03 | -1.18 |
| Corticosteroid 11-beta-dehydrogenase isozyme 1 (Hsd11b1) | 5.73E-03 | 1.24 | 3.01E-03 | 1.39 |
