# Supplementary figures and images for: Polyomic profiling reveals significant hepatic metabolic alterations in glucagon-receptor (GCGR) knockout mice: implications on anti-glucagon therapies for diabetes
Source: BMC Genomics. 2011 Jun 1;12:281. doi: 10.1186/1471-2164-12-281 (PMC3130710; doi:10.1186/1471-2164-12-281)

**Additional file 3**: Correlation analysis between mRNA and Protein Changes


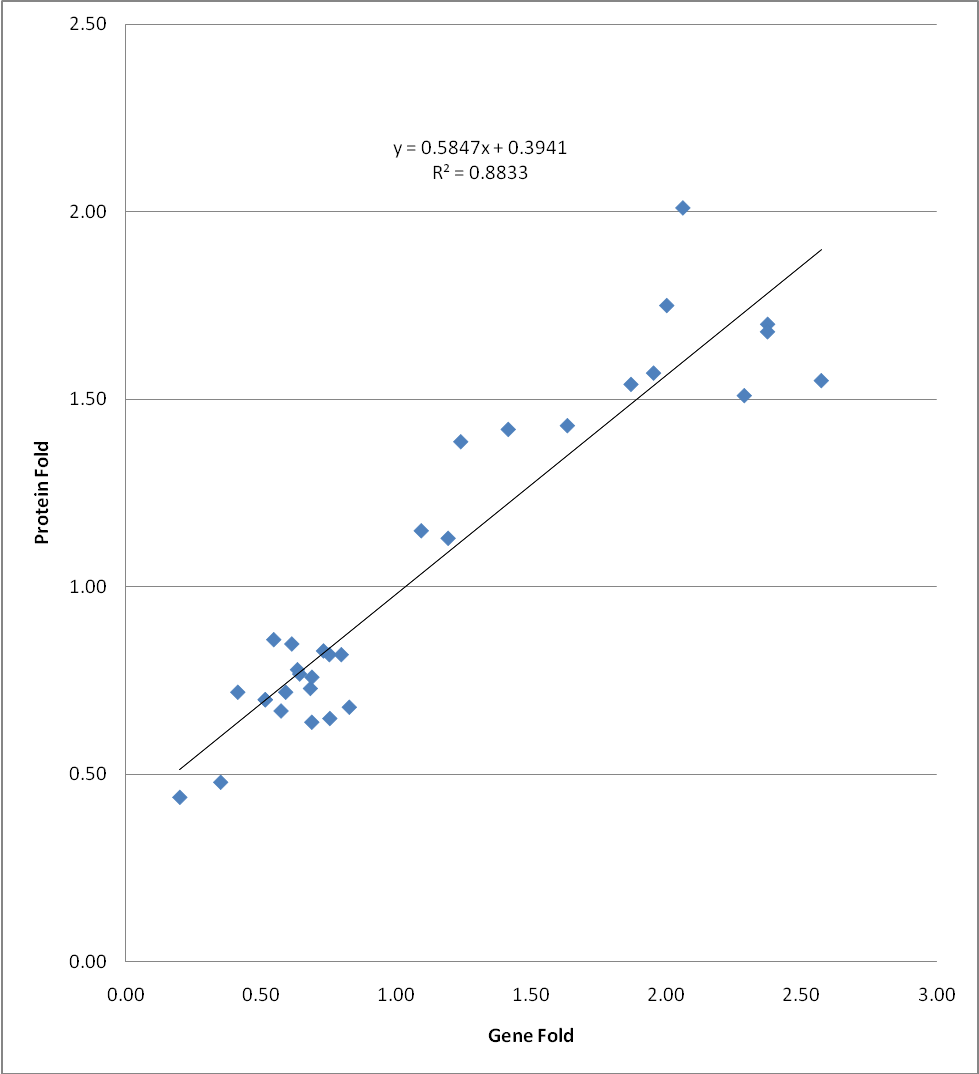

Supplement: Additional file 3 — Correlation analysis between mRNA and Protein Changes. Linear regression analysis demonstrated correlation between mRNA and protein expression changes [file 1471-2164-12-281-S3.DOC]
