# Supplementary material for: Polyomic profiling reveals significant hepatic metabolic alterations in glucagon-receptor (GCGR) knockout mice: implications on anti-glucagon therapies for diabetes
Source: BMC Genomics. 2011 Jun 1;12:281. doi: 10.1186/1471-2164-12-281 (PMC3130710; doi:10.1186/1471-2164-12-281)
Supplement: Additional file 4 — Significantly Changed mRNA related to Protein Translation. The table summarized significantly changed mRNA related to protein translation in the GCGR KO liver. [file 1471-2164-12-281-S4.DOC]

Additional file 4: Significantly Changed mRNA related to Protein Translation

| **Gene** | Adj. p-value | Fold change |
| --- | --- | --- |
| Insulin-like growth factor 1 (Igf1) | 0.0072 | -1.41 |
| DnaJ (Hsp40) homolog, subfamily C, member 1 (Dnajc1) | 0.0134 | -1.37 |
| Alanyl-tRNA synthetase (Aars) | 0.0088 | -1.30 |
| Mitogen-activated protein kinase 1 (Mapk1) | 0.0133 | -1.21 |
| Methyltransferase 11 domain containing 1 (Mett11d1) | 0.0132 | -1.21 |
| Mitochondrial ribosomal protein L44 (Mrpl44) | 0.0113 | -1.20 |
| Mitchondrial ribosomal protein S7 (Mrps7) | 0.0159 | -1.13 |
| Ribosomal protein L41 (Rpl41) | 0.0105 | 1.12 |
| Ribosomal protein L15 (Rpl15) | 0.0146 | 1.12 |
| Ribosomal protein 10 (Rpl10) | 0.0096 | 1.13 |
| Ribosomal protein L19 (Rpl19) | 0.0073 | 1.14 |
| Mitochondrial ribosomal protein L48 (Mrpl48) | 0.0095 | 1.16 |
| Eukaryotic translation elongation factor 1 gamma (Eef1g) | 0.0135 | 1.17 |
| Ribosomal protein S14 (Rps14) | 0.0163 | 1.18 |
| Ribosomal protein L27 (Rpl27) | 0.0155 | 1.19 |
| Eukaryotic translation elongation factor 1 delta (Eef1d) | 0.0126 | 1.20 |
| Ribosomal protein L7A (Rpl7a) | 0.0087 | 1.20 |
| Ribosomal protein L21 (Rpl21) | 0.0066 | 1.20 |
| Ribosomal protein L26 (Rpl26) | 0.0091 | 1.20 |
| Ribosomal protein S3 (Rps3) | 0.0125 | 1.20 |
| Ribosomal protein S26 (Rps26) | 0.0177 | 1.20 |
| Eukaryotic translation initiation factor 3, subunit H (Eif3h) | 0.0090 | 1.20 |
| Ribosomal protein S25 (Rps25) | 0.0166 | 1.21 |
| Ribosomal protein L18A (Rpl18a) | 0.0086 | 1.21 |
| Ribosomal protein L22 (Rpl22) | 0.0037 | 1.21 |
| Ribosomal protein L39 (Rpl39) | 0.0177 | 1.21 |
| Ribosomal protein L18 (Rpl18) | 0.0102 | 1.21 |
| Ribosomal protein L23a (Rpl23a) | 0.0132 | 1.22 |
| Ribosomal protein L13A (Rpl13a) | 0.0165 | 1.22 |
| Eukaryotic translation initiation factor 3, subunit E (Eif3e) | 0.0112 | 1.23 |
| Ribosomal protein S21 (Rps21) | 0.0099 | 1.23 |
| Ribosomal protein S24 (Rps24) | 0.0170 | 1.23 |
| Ribosomal protein S20 (Rps20) | 0.0112 | 1.23 |
| Eukaryotic translation initiation factor 2, subunit 3 (Eif2s3x) | 0.0162 | 1.26 |
| Ribosomal protein S9 (Rps9) | 0.0184 | 1.26 |
| Ribosomal protein L11 (Rpl11) | 0.0032 | 1.26 |
| Ribosomal protein S10 (Rps10) | 0.0081 | 1.26 |
| Eukaryotic translation elongation factor 1 beta 2 (Eef1b2) | 0.0126 | 1.27 |
